# Artepillin C-rich propolis extract supplementation promotes muscle recovery following exercise-induced muscle damage in resistance-trained young females: a randomized, placebo-controlled trial

**DOI:** 10.1080/15502783.2025.2569908

**Published:** 2025-10-11

**Authors:** Olavo João Frederico Ramos Junior, Natália Soares Veiga, Andresa Aparecida Berretta, Thiago Silveira Alvares

**Affiliations:** a Federal University of Rio de Janeiro, Nutrition and Exercise Metabolism Research Group, Multidisciplinary Center UFRJ-Macaé, Macaé City, Brazil; b Federal University of Rio de Janeiro, Multicenter Graduate Program in Physiological Sciences, Macaé City, Brazil; c Apis Flora Indl. Coml. Ltda, Research, Development & Innovation Laboratory, Ribeirão Preto, Brazil; d Federal University of Rio de Janeiro, Food and Nutrition Institute, Multidisciplinary Center UFRJ-Macaé, Macaé City, Brazil

**Keywords:** Nutraceuticals, polyphenols, antioxidants, artepillin C, muscle recovery, echo intensity

## Abstract

**Introduction:**

Exercise-induced muscle damage (EIMD) results from intense or unaccustomed exercise, leading to inflammation, oxidative stress, and reduced muscle function due to excessive reactive oxygen species. Propolis, a natural bee-derived substance rich in bioactive coumpounds such as artepillin-c, exhibits anti-inflammatory and antioxidant properties, suggesting its potential to mitigate EIMD. This study investigated the effect of a standardized Brazilian green propolis extract (EPP-AF) on recovery from EIMD, which is characterized by muscle soreness, reduced function, and lower muscle quality.

**Methods:**

Twenty-two trained female participants were randomly assigned to consume eight capsules of EPP-AF (containing approximately 54 mg artepillin C) or a placebo (PLA) for seven days. On day four, participants performed 10 sets of 10 maximal eccentric contractions of the knee extensor muscles. Maximal voluntary isokinetic torque (MVIT), muscle thickness (MT), muscle ultrasound echo intensity (EI), and delayed onset muscle soreness (DOMS) were assessed before and at 2 h, 24 h, 48 h, and 72 h post-EIMD.

**Results:**

The EIMD protocol significantly increased MT (*p* = 0.031), EI (*p* = 0.013), and DOMS (*p* < 0.001) while reducing MVIT (*p* < 0.001). Compared to placebo, EPP-AF supplementation attenuated DOMS (*p* < 0.001), mitigated increases in MT (*p* = 0.025) and EI (*p* = 0.043), and accelerated MVIT recovery (*p* = 0.037) in the days following days of eccentric exercise compared to placebo.

**Conclusion:**

EPP-AF may alleviate the symptoms and attenuate markers of muscle damage in the knee extensor muscles of resistance-trained females. These findings highlight the potential of propolis as a natural intervention to enhance recovery from EIMD.

## 1. Introduction

Exercise-induced muscle damage (EIMD) is characterized by the disruption of cellular membranes, mitochondrial dysfunction, increased oxidative stress, inflammation, and impaired muscle performance, which may persist for several days post-exercise [1]. EIMD typically arises from training protocols with high volume, high intensity, or a predominance of eccentric muscle actions, especially when recovery is insufficient [2]. In addition, prolonged and intense exercise exacerbates oxidative stress due to the elevated production of reactive oxygen and nitrogen species by contracting skeletal muscles. This accumulation can induce oxidative damage to intracellular proteins and lipids, thereby impairing contractile function, reducing force generation, and contributing to muscle weakness [3,4].

Activities such as downhill running, plyometric training, and resistance exercises frequently involve repeated eccentric contractions and are commonly performed by the general population [5]. Although EIMD represents a natural physiological stimulus essential for muscle adaptation and long-term improvements in strength and function [6], its acute manifestations – such as muscle soreness, reduced range of motion, and residual discomfort – can impair training progression and performance. These symptoms may persist for several days, depending on the extent of the damage [2,6]. Therefore, nutritional strategies aimed at attenuating the transient negative effects of EIMD may be especially valuable for trained individuals with limited recovery periods between training sessions or competitive events [7,8].

Nutritional interventions containing bioactive compounds rich in phytochemicals – such as curcumin [9,10], jaboticaba berries [11], and pomegranate [12]—have been shown to be beneficial in promoting recovery from muscle damage. In addition, another promising compound found in Brazilian propolis has been reported to exhibit antioxidant and anti-inflammatory properties in both *in vitro* [13–15] and *in vivo* [16–18]. Brazilian Green propolis extract from *Baccharis dracunculifolia*, produced by *Apis mellifera* honeybees, contains biologically active constituents, including a wide variety of organic compounds [19]. The primary bioactive compounds responsible for the biological and nutraceutical properties are prenylated cinnamic acid derivatives, such as artepillin C [20,21]. Their mechanisms of action are thought to involve inhibition of the NF-κB signaling pathway and suppression of inflammasome activation [22,23].

Although the health benefits of propolis are well-established, limited research has explored its effects in the context of exercise. Existing studies have primarily focused on its impact on performance (e.g. VO₂ max and fatigue index) in healthy males [24], metabolic marks (e.g. interleukin-6, superoxide dismutase, malondialdehyde, and total antioxidant capacity) in females with diabetic dyslipidemia [25], and patients with nonalcoholic fatty liver disease [26]. To our knowledge, no study has specifically investigated the effects of propolis supplementation on muscle recovery following EIMD in resistance-trained females. This population experiences hormonal fluctuations (e.g. estrogen) that may affect muscle recovery and associated symptoms, such as the perception of muscle soreness following EIMD [27].

Therefore, the present study aimed to evaluate the effects of a seven-day supplementation with a standardized Brazilian green propolis extract on markers of muscle damage and morphological muscle quality in the knee extensor muscles following eccentric exercise in resistance-trained females. We hypothesized that propolis extract would attenuate oxidative stress and the associated muscle damage, thereby accelerating the recovery of the knee extensor muscles after EIMD compared to placebo.

## 2. Method

### 2.1. Participants

Twenty-two trained female participants – a population that may be more susceptible to exercise-induced muscle damage – were recruited through social media and advertisements at the Federal University of Rio de Janeiro, Macaé, Brazil. The inclusion criteria were females aged between 18 and 35 years with at least three months of consistent resistance training experience (minimum three sessions per week). Exclusion criteria included the presence of chronic diseases (e.g. diabetes mellitus, hypertension, heart failure), smoking, pregnancy or breastfeeding status, injuries to the upper or lower limbs, and under the use of nutritional supplements (e.g. creatine, caffeine, and antioxidant vitamins), anabolic steroids, or anti-inflammatory medications. Baseline characteristics of the participants are summarized in Table 1.

**Table 1.** Baseline characteristics of the participants.

	PLA (n = 11)	EPP-AF (n = 11)
Age (years)	24 ± 5	22 ± 1.4
Weight (kg)	56.6 ± 7.6	57.9 ± 8.9
Height (m)	1.60 ± 4.6	1.59 ± 0.05
BMI (kg/m^2^)	22.6 ± 2.7	22.5 ± 2.4
MVIT (Nm.kg^−1^)	3.60 ± 0.7	3.53 ± 0.5
ATT (cm)	3.94 ± 0.7	4.05 ± 0.6

Values are expressed as mean ± SD. ATT = adipose tissue thickness; BMI = body mass index; EPP-AF = Standardized Brazilian green propolis extract; MVIT = maximal voluntary isokinetic torque; PLA = placebo group.

The sample size was determined based on the primary outcome (i.e. muscle strength recovery). An a priori power analysis was conducted using G*Power version 3.1.9 for a two-way ANOVA with repeated measures and within-between interaction. With a target statistical power (1 – β) of 0.80, a moderate effect size (Cohen’s *f*) of 0.25, and a significance level set at 0.05, a minimum of twenty-two participants was required to prevent a type 2 statistical error. All experimental procedures were conducted in accordance with the ethical principles outlined in the Declaration of Helsinki and received approval from the Institutional Ethics Committee of the Federal University of Rio de Janeiro – Macaé Campus, Rio de Janeiro, Brazil (protocol CAAE: 57,146,722.10000.5699). All participants were fully informed of the study’s objectives and provided written informed consent prior to participation.

### 2.2. Experimental design

This study was a randomized, double-blind, placebo-controlled trial with a parallel-group design. Participants were assigned to either the standardized green Brazilian green propolis extract (EPP-AF) or placebo (PLA) group using a counterbalanced randomization method based on their maximum isokinetic torque. In this double-blind study, both participants and outcome assessors were unaware of group allocation. Supplementation was coded and distributed by an independent researcher, and both capsules were identical in appearance. At the beginning of the study, participants attended a familiarization session where anthropometric measurements were taken, and they performed one set of exercises used to induce muscle damage and assess maximal strength. During this session, they were randomly assigned to receive either EPP-AF or PLA.

Participants visited the laboratory over five days. On day 1, baseline testing and familiarization with isokinetic exercise protocols were conducted. One week after familiarization, participants began supplementation, taking four capsules of either the EPP-AF or PLA twice daily for seven days (three days before exercise-induced muscle damage [EIMD], on the day of eccentric exercise, and three days post-exercise).

**Figure 1.**
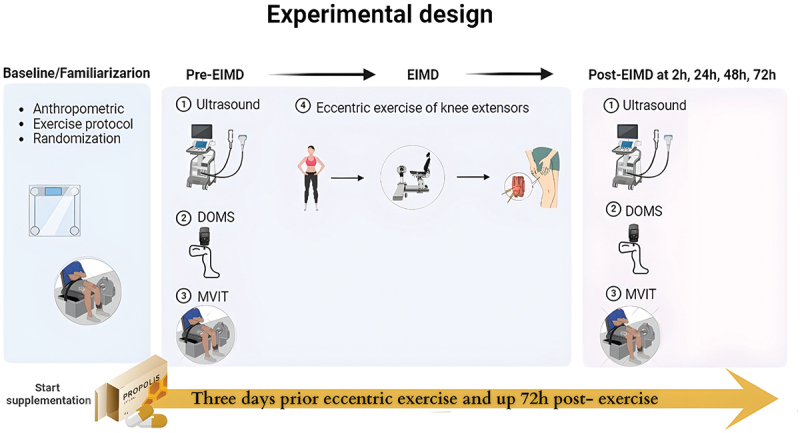
Timeline of study protocol from familiarization to 72 h post-exercise. EPP-AF = standardized Brazilian green propolis extract; PLA = placebo; DOMS = delayed onset muscle soreness; EIMD = exercise-induced muscle damage; MVIT = maximal voluntary isokinetic torque.

On day 4, participants returned to the laboratory after an overnight fast. Pre-EIMD measurements included maximal voluntary isokinetic torque (MVIT), muscle soreness, and ultrasound measurements of muscle thickness (MT) and echo intensity (EI). Participants then consumed their morning dose of EPP-AF or PLA, waited for one hour, and performed the EIMD protocol. The same pre-exercise assessments (MVIT, muscle soreness, MT, and EI) were repeated at 2 h, 24 h (day 5), 48 h (day 6), and 72 h (day 7) post-EIMD protocol (Figure 1). The 2-hour time point was selected instead of immediate post-exercise assessment to minimize the influence of acute neuromuscular fatigue and better capture early markers of muscle damage [28,29].

All participants were instructed to refrain from any exercise for 48 h before day 4 and throughout the study period (until day 7). To account for menstrual cycle variations, female participants were tested to the end of the luteal phase and the onset of menstruation, as lower estrogen levels during this period may influence perceptions of force and muscle soreness [30].

### 2.3. Dietary control

Participants were instructed to avoid polyphenol-rich foods and beverages – including propolis, alcohol, caffeine, and antioxidant supplements – for 48 hours before each session and throughout the recovery period, to minimize dietary interference with recovery and inflammation markers. The list of restricted items (Supplementary Table S1) was adapted from previous literature identifying high-polyphenol foods [31,32]. To facilitate adherence, a detailed list of foods and food groups to avoid, as well as recommended alternatives, was provided. Compliance with the dietary restrictions was monitored using a 24-hour dietary recall, which allowed for the estimation of daily polyphenol intake during the study period.

### 2.4. Nutritional supplementation

For the purposes of this study and aiming avoid the differences that can appear due to the quali and quantitative differences in the chemical and biological activities, it was used the standardized green propolis extract EPP-AF® [33], that was already studied under the safety and biological aspects since in vitro until in clinical trials [34–36].

A standardized Brazilian green propolis extract (EPP-AF®, Apis Flora Indl. Coml. Ltda., Ribeirão Preto, São Paulo State, Brazil) was used in the present study, with the studied batch containing approximately 54 mg artepillin C per dose. This amount was chosen based on previous studies demonstrating antioxidant and anti-inflammatory effects after consumption of EPP-AF [34–36], Although these studies did not specifically investigate muscle damage, they reported modulation of key biological pathways involved in EIMD – such as inhibition of NF-κB signaling, suppression of inflammasome activation, and reduction of pro-inflammatory cytokine production (e.g. IL-1β, IL-6, TNF-α) [22,23,37]. These mechanisms are central to secondary muscle damage and delayed recovery after eccentric exercise. Artepillin C is a prenylated phenolic compound uniquely found in Brazilian green propolis and is recognized as the principal bioactive marker of EPP-AF®. Its presence ensures chemical standardization and reproducibility and is thought to underlie the extract’s biological activity. The placebo (PLA), composed of corn starch, was matched to EPP-AF in color, texture, taste, and packaging, and contained no propolis-derived compounds.

Participants in both the EPP-AF and PLA groups were instructed to consume four capsules twice daily (eight capsules total) for seven days. Adherence to the intervention and any potential adverse effects were monitored through participant feedback. Artepillin C in EPP-AF was determined by UV detection using a high-performance liquid chromatography system [33].

Dietary intake of phenolic acids, flavonoids, antioxidant vitamins (e.g. ascorbic acid, tocopherol, retinol), macronutrients, and total energy was assessed throughout the EPP-AF supplementation period using repeated 24-hour dietary recalls. Participants reported the type and amount of foods consumed daily, which were then converted into gram equivalents using standard household measures. The nutritional composition was analyzed using the WebDiet software (version 2.0, Rio de Janeiro, RJ, Brazil), with reference values obtained from the Brazilian Food Composition Table [38].

### 2.5. Exercise-induced muscle damage protocol

Before the eccentric exercise, participants performed a 2-minute warm-up consisting of dynamic knee extension and flexion movements using the isokinetic dynamometer. The eccentric exercise protocol consisted of 10 sets of 10 maximal eccentric contractions of the non-dominant knee extensors using an isokinetic dynamometer (Humac Norm, CSMi Medical Solutions, MA, USA). Each repetition began with a passive knee extension from 90° to 0° at 120°·s^−1^. This was immediately followed by an eccentric knee flexion phase, during which the dynamometer moved the limb from 0° to 90° at 30°·s^−1^. Participants were instructed to maximally resist this movement, resulting in eccentric loading of the quadriceps muscles. A 1-minute rest interval was provided between sets.

### 2.6. Muscle strength assessment

Muscle strength was assessed as maximal voluntary isokinetic torque (MVIT) using an isokinetic dynamometer (Humac Norm, CSMi Medical Solutions, MA, USA). Participants performed four maximal isokinetic contractions of the non-dominant knee involving both extension and flexion movements. However, peak torque was calculated only during the extension phase, which corresponds to the active contraction of the knee extensor muscles (quadriceps), the primary muscle group targeted by the EIMD protocol. The extension phase was performed at an angular velocity of 60°·s^−1^, while the return flexion phase was conducted passively at 120°·s^−1^ to allow a smooth repositioning of the limb. The highest peak torque value recorded during the active contractions was used for data analysis. This procedure was conducted on day 4 (pre-EIMD) and repeated at 2 h, 24 h, 48 h, and 72 h after the EIMD protocol. Absolute MVIT values were converted to relative values (% change from pre-EIMD) for statistical analysis.

### 2.7. Muscle thickness and echo intensity

Changes in the non-dominant knee extensors muscle thickness (MT) before and in the following days of EIMD were measured using B-mode ultrasonography (LOGIC*e*, GE HealthCare, Chicago, IL, USA) with a 5.0–10.0 MHz linear-array probe. Participants rested in the supine position for 10 minutes before images were acquired. The ultrasound probe was coated with a water-soluble gel and positioned transversely and perpendicularly over the measuring point on the anterior surface of the leg, where vastus intermedius and rectus femoris muscle thickness (MT) was recorded. The muscle tissue interface between the bone and adipose tissue was identified, and the image on the monitor was frozen. With the image frozen, a cursor was enabled to measure MT. Three MT measures were taken and averaged to achieve a final MT value. The same person performed the ultrasound image and measuring point identification in all testing visits.

Morphological muscle quality (MQ_m_) refers to intramuscular changes in muscle architecture and composition and was assessed on an ultrasound device using post hoc analysis of density by quantifying echo intensity (EI) [39]. The EI was quantified using a gray-scale analysis function and expressed in arbitrary units (a.u.) as a value between 0 (black) and 255 (white) using ImageJ software (National Institute of Health, Bethesda, USA, Version 1.37). The increase of EI (i.e. brighter image) in response to exercise has been suggested to represent muscle damage and inflammation [40]. Corrected EI was calculated as uncorrected EI + (subcutaneous fat thickness [cm] × 40.5278) [41].

To ensure the reliability and consistency of ultrasound assessments, all measurements were performed by the same trained and experienced evaluator using fixed device settings (depth = 4.0 cm; gain = 60 dB). The anatomical measurement site was standardized as the midpoint between the anterior superior iliac spine and the superior border of the patella, measured with a tape measure [42]. This point was marked with a dermatological pen to assist in transducer positioning across all time points. Intraday reliability testing conducted in our laboratory using the same evaluator and equipment yielded excellent reproducibility: coefficients of variation (CV) were 0.3% for muscle thickness and 0.5% for echo intensity, while intraclass correlation coefficients (ICC) were 0.999 and 0.998, respectively. Additionally, representative pre- and post-exercise ultrasound images for each group are presented in Figure X to illustrate changes in muscle architecture and echo intensity following the eccentric exercise protocol.

### 2.8. Muscle soreness

Muscle pain sensitivity was measured using a digital pressure algometer equipped with a 1.0 cm^2^ probe. The reliability and validity of this device have been established in previous research [43]. Pressure pain threshold was used as an objective proxy for DOMS, as it quantifies local mechanical sensitivity that typically decreases after eccentric exercise due to muscle damage and inflammation, and has been widely applied in prior studies [44,45]. Pressure sensitivity assessments were conducted on the rectus femoris, vastus lateralis, and vastus medialis muscles of the non-dominant leg. A trained and experienced researcher performed the measurements by applying pressure at the approximate midpoint of each muscle using the probe. Participants were instructed to indicate when they felt pain or discomfort, known as the pressure pain threshold, and the corresponding force value (in kilograms) was recorded. Each measurement was taken in triplicate on the dominant thigh while participants were seated with their knees positioned at a 90° angle. Prior to these assessments, a familiarization session was conducted on the non-dominant leg. The measurements were repeated before and after 2 h, 24 h, 48 h, and 72 h following the EIMD protocol.

### 2.9. Statistical analysis

A two-way mixed ANOVA with repeated measures (2 × 5; group × time; between-within subject design) was performed to determine the differences in maximal voluntary isokinetic torque (MVIT), muscle thickness (MT), echo intensity (EI), and delayed onset muscle soreness (DOMS) between the EPP-AF and PLA groups. When the F test indicated a rejection of the null hypothesis, a multiple comparison test was carried out using the Bonferroni adjustment. The effect size was calculated using Cohen’s partial eta squared. All statistical analyses were performed using IBM SPSS Statistics version 27.0 (SPSS Inc., Chicago, IL, USA), with the significance level (α) set at 0.05. Data are presented as mean ± standard deviation.

## 3. Results

All twenty-two participants completed the study and confirmed full adherence to the supplementation protocol. The EPP-AF provided in this study was well tolerated, with no reported side effects. Additionally, there were no statistically significant differences between groups in the intake of energy, macronutrients (carbohydrates, protein, and fats), antioxidant vitamins (vitamin A, vitamin C, and vitamin E), phenolic acids, and flavonoids throughout the study period (Table 2).

**Table 2.** Estimated energy and nutrient intake from 24-hour dietary recalls over the study period.

Nutrient	EPP-AF					PLA				
	Baseline	Pre-EIMD	24 h	48 h	72 h	Baseline	Pre-EIMD	24 h	48 h	72 h
Energy (kcal)	1696.1 ± 246.2	1857.5 ± 351.2	1707.8 ± 299.8	1730.2 ± 336.1	1770.8 ± 359.4	1784.3 ± 401.7	1753.5 ± 378.6	1729.3 ± 336.1	1711.9 ± 218.3	1848.8 ± 282.9
Carbohydrates (g)	172.5 ± 49.9	217.7 ± 67.1	188.5 ± 42.8	196.1 ± 67.9	210.2 ± 65.2	187.1 ± 42.1	192.4 ± 77.8	182.1 ± 37.1	191.4 ± 43.5	207.4 ± 24.7
Proteins (g)	113.8 ± 22.4	117.9 ± 30.5	100.1 ± 41.4	95.4 ± 34.7	99 ± 28.6	112.1 ± 40.7	109.9 ± 29.2	115.7 ± 31.9	118.7 ± 30.8	114.4 ± 37.4
Lipids (g)	69.1 ± 10.9	65.3 ± 16.2	61.7 ± 16.8	62.8 ± 20.5	62.1 ± 21.4	68.7 ± 20.6	65.2 ± 18.9	58.2 ± 14.6	61.5 ± 14.9	68.5 ± 22.4
Retinol (mcg)	249.7 ± 80.4	236.4 ± 96.4	243.7 ± 64.1	314.7 ± 88.7	266.72 ± 82.3	259.2 ± 112.21	188.2 ± 85.9	244.7 ± 89.1	269.2 ± 72.5	260.3 ± 64.3
Ascorbic acid (mg)	203.5 ± 33.9	186.9 ± 76	194.1 ± 87.6	229.3 ± 46.1	221.3 ± 53.9	204.3 ± 78.8	209.4 ± 71.9	186.8 ± 69.1	216.2 ± 53.7	206.6 ± 94.5
Tocopherol (mg)	7.2 ± 3.3	8.1 ± 2.5	7.1 ± 3.6	7.5 ± 3.9	8.1 ± 3.9	7.9 ± 4.9	9.9 ± 3.3	9.4 ± 3.2	8.4 ± 2.8	10.4 ± 3.8
Phenolic Acids (mg)	226.69 ± 93.8	206.78 ± 88.6	269.75 ± 76.4	202.35 ± 79.2	237.64 ± 92.4	285.5 ± 52.9	263.2 ± 91.2	245.2 ± 89.3	281.7 ± 62.5	282.7 ± 82.87.9
Flavonoids (mg)	64.50 ± 9.1	127.61 ± 84.5	116.76 ± 35.5	115.69 ± 59.9	129.86 ± 83.7	44.2 ± 6.6	111.86 ± 33.7	113.34 ± 83.4	102.4 ± 50.4	112.6 ± 51.8

Values are mean ± standard deviation. EIMD = exercise-induced muscle damage; EPP-AF = standardized Brazilian green propolis extract; PLA = placebo group. Nutrients from EPP-AF were not included in the table.

### 3.1. Muscle strength

**Figure 2.**
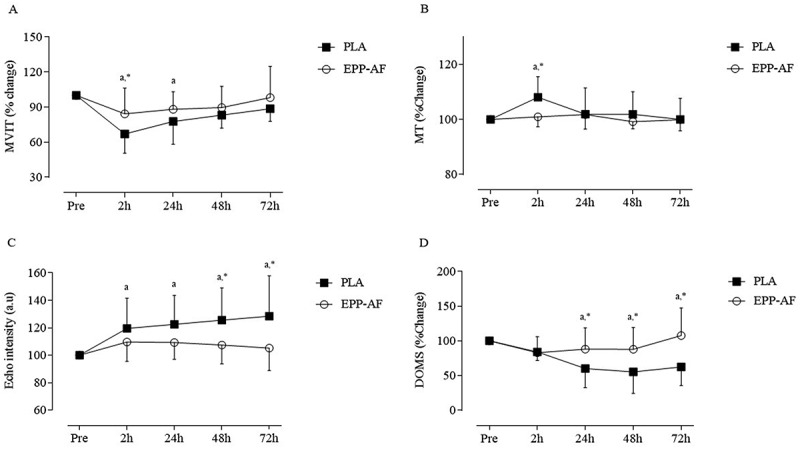
Changes in (A) Maximal voluntary isokinetic torque (MVIT), (B) Muscle thickness (MT), (C) Morphological muscle quality (MQ_m_) and (D) delayed onset muscle soreness (DOMS) at pre and 2 h, 24 h, 48 h, and 72 h following exercise-induced muscle damage (EIMD) protocol in both standardized Brazilian green propolis extract (EPP-AF) and control (PLA) groups. The letter ^a^denotes a significant difference (*p* < 0.05) from pre values for both EPP-AF and PLA groups. The symbol * denotes a significant difference (*p* < 0.05) between groups. Values are expressed as mean ± SD.

Changes in MVIT between EPP-AF and PLA groups after the EIMD are detailed in Table 3 and Figure 2A. All participants experienced a decrease in MVIT of at least 20% compared to their pre-damage measurements. A significant main effect for time was observed for MVIT (*p* < 0.001, η2_p =_ 0.401). Post hoc analysis revealed a significant decrease in MVIT at 2 h (*p* < 0.001) and 24 h (*p* < 0.029) in the PLA group following the EIMD when compared to pre-exercise values. Furthermore, a significant interaction effect was observed regarding supplementation per time (*p* = 0.048, η2_p =_ 0.398). Post hoc analysis revealed a significant difference in MVIT between EPP-AF and PLA groups at 2 h (*p* = 0.037) following the EIMD.

**Table 3.** Changes in muscle strength, muscle thickness, echo intensity, and perceived muscle soreness in the standardized Brazilian green propolis (EPP-AF) and placebo (PLA) groups at baseline (pre) and over 72 hours following exercise-induced muscle damage.

Variable	Group	Pre-EIMD	2 h	24 h	48 h	72 h	*p-value* *(interaction)*
MVIT (Nm.kg)	EPP-AF	2.50 ± 0.48	1.97 ± 0.57	2.09 ± 0.61	2.11 ± 0.79	2.24 ± 0.71	0.245
	PLA	2.32 ± 0.67	1.40 ± 0.66	1.97 ± 0.56	2.06 ± 0.54	2.21 ± 0.52	
MVIT (% change)	EPP-AF	100	84.29 ± 22.10	88.19 ± 14.96	89.58 ± 18.22	98.16 ± 26.61	0.037
	PLA	100	64.51 ± 14.66^a,*^	80.18 ± 19.20^a^	86.15 ± 8.9	90.99 ± 8.8	
MT (cm)	EPP-AF	4.05 ±0.65	4.04 ± 0.65	4.05 ± 0.58	4.11 ± 0.53	4.13 ± 0.45	0.239
	PLA	4.16 ± 0.49	4.37 ±0.61^a,^	4.27 ± 0.74	4.30 ± 0.85	4.17 ± 0.51	
MT (% change)	EPP-AF	100	100.92 ± 3.58	101.80 ± 5.28	99.16 ± 2.58	99.86 ± 4	0.025
	PLA	100	108.06 ± 7.53^a,*^	101.91 ± 9.57	101.85 ± 8.20	100.04 ± 7.6	
EI (a.u)	EPP-AF	65.69 ±12.84	74.87 ± 7.11	77.29 ± 6.41	75.86 ± 9.56	79.84 ± 13.63	0.286
	PLA	66.56 ± 14.26	78.01 ± 9.61	79.05 ± 27.83	87.98 ± 14.51	90.66 ± 25.40	
EI (% change)	EPP-AF	100	109.67 ±14.14	109.30 ± 12.21	107.42 ± 13.62	105.25 ± 16.37	0.043
	PLA	100	119.58 ± 21.95^a^	122.53 ± 21.15^a^	125.57 ± 23.55^a,*^	128.51 ± 29.43^a,*^	
DOMS (N.cm^2^)	EPP-AF	4.62 ± 0.74	4.34 ± 0.23	4.84 ± 1.04	4.86 ± 0.94	5.12 ± 1.01	0.371
	PLA	4.79 ± 3.04	3.89 ± 2.26	3.92 ± 2.08^a^	4.17 ± 2.41^a^	4.45 ± 2.84^a^	
DOMS (% change)	EPP-AF	100	82.82 ± 23.23	87.98 ± 30.77	87.91 ± 31.21	107.66 ± 39.67	0.004
	PLA	100	83.97 ±12.29	60.09 ± 27.66^a,*^	55.32 ± 30.92^a,*^	62.30 ± 26.58*	

Data are mean ± standard deviation. DOMS = delayed-onset muscle soreness; EIMD = exercise-induced muscle damage; EPP-AF = standardized Brazilian green propolis extract; MT = muscle thickness; EI = echo intensity; MVIT = maximal voluntary isokinetic torque; % change = percentage change from Pre-EIMD. The symbol * denotes a significant difference (*p* < 0.05) between groups. A repeated measures two-way ANOVA was used to identify differences in the variables between the EPP-AF and PLA groups. Absolute data were converted to relative values and presented as % change from Pre-EIMD.

### 3.2. Muscle thickness

Changes in MT between EPP-AF and PLA groups are presented in Table 3 and Figure 2B. A significant main effect for time was observed for MT (*p* = 0.031, η2_p =_ 0.390). Post hoc analysis indicated a significant increase in MT at 2 h following EIMD only in PLA group (*p* < 0.038). Furthermore, there was a significant interaction, and post hoc analysis revealed a significant difference in MT between the EPP-AF and control groups at 2 h (*p* = 0.025, η2_p =_ 0.221) following EIMD.

### 3.3. Morphological muscle quality

Changes in echo intensity between the EPP-AF and PLA groups after the EIMD are presented in Table 3 and Figure 2C. A significant main effect for time was observed (*p* = 0.017, η2_p =_ 0.472). Post hoc analysis demonstrated a significant increase in knee extensors muscle echo intensity in PLA group at 2 h (*p* = 0.035), 24 h (*p* = 0.006), 48 h (*p* = 0.005), and 72 h (*p* = 0.014) following the EIMD compared to pre-exercise values. Furthermore, a significant interaction effect regarding supplementation per time was found (*p* = 0.049, η2_p =_ 0.364). The post hoc analysis revealed significant differences in EI between EPP-AF and PLA groups at 48 h (*p* = 0.049) and 72 h (*p* = 0.043) following the EIMD.

### 3.4. Muscle soreness

Changes in perceived muscle soreness between EPP-AF and PLA groups after EIMD are illustrated in Table 3 and Figure 2D. A significant main effect for time was observed for DOMS (*p* < 0.001, η2_p =_ 0.483). Post hoc analysis indicated a significant increase in perceived muscle soreness in the PLA group at 24 h (*p* = 0.016), 48 h (*p* = 0.010), and 72 h (*p* = 0.049) following the EIMD compared to pre-exercise values. Additionally, a significant interaction effect regarding supplementation per time was observed (*p* = 0.001, η2_p =_ 0.393). The post hoc analysis revealed significant differences in DOMS between EPP-AF and PLA groups at 24 h (*p* = 0.049), 48 h (*p* = 0.048), and 72 h (*p* = 0.015) following the EIMD.

## 4. Discussion

This study is the first to examine the effects of artepillin C-rich propolis extract supplementation on the recovery of skeletal muscle following eccentric exercise-induced muscle damage in resistance-trained females. The eccentric exercise protocol employed in the present study has been used in prior studies [12,46] and was chosen to induce physiological changes associated with muscle damage, allowing for the assessment of the bioactive compounds in propolis extract on muscle recovery days after eccentric exercise.

The primary findings of the present study demonstrate that propolis extract supplementation improved the recovery of: (1) muscle strength; muscle thickness; (3) morphological muscle quality and (4) muscle soreness. These results support the potential of propolis, due to its antioxidant and anti-inflammatory properties [24,37], to enhance post-exercise recovery in physically active individuals.

Furthermore, a previous study has shown that propolis intake increases blood levels of artepillin C, which can exert biological effects [21]. Circulating artepillin C levels has been demonstrated to be higher in females than in males, suggesting that daily propolis intake may be particularly effective in females [47]. This difference may be due to sex-related variations in polyphenol metabolism and absorption, as shown by Ding et al. [47], who reported distinct artepillin C pharmacokinetic profiles between males and females. While the mechanisms remain unclear, these variations appear to be compound-specific and warrant further investigation, especially in long-term studies and physically active populations. This supports the hypothesis that propolis extract may accelerate muscle recovery while reducing oxidative stress and inflammation after EIMD in resistance-trained females.

Eccentric exercise resulted in significant increases in indirect markers of muscle damage, including muscle soreness, decreased isokinetic peak torque, reduced muscle quality (echo intensity), and elevated CK levels. However, EPP-AF supplementation attenuated several of these responses. Notably, participants in the EPP-AF group showed faster recovery of muscle function and quality, and reported less muscle soreness compared to placebo, suggesting a protective or restorative effect of the propolis extract.

These effects may be explained by the known biological properties of EPP-AF, which is rich in phenolic compounds such as artepillin C, baccharin, and drupanin. These compounds possess strong antioxidant and anti-inflammatory activity [34–36,48]. Previous studies have shown that phenolics from propolis can modulate inflammatory pathways by reducing neutrophil infiltration, suppressing NF-κB activation, and inhibiting the expression of COX-2 and iNOS, as well as cytokine production (e.g. IL-1β, MCP-1) [22,23,49]. Such actions may limit the secondary inflammatory cascade commonly associated with muscle damage, thereby facilitating recovery.

Regarding oxidative stress, phenolic compounds in propolis have been positively associated with total antioxidant capacity (as measured by FRAP and ORAC assays) [50]. In preclinical models, artepillin C has been shown to reduce SOD activity and enhance GST and CAT activity [17], supporting its role in ROS detoxification. These antioxidant effects may help mitigate oxidative damage triggered by eccentric contractions, which is known to contribute to impaired muscle function, delayed recovery, and increased muscle soreness [51–53].

Interestingly, Soleimani et al. [24] evaluate the effect of supplementing 450 mg of propolis in tablet form (containing 180 mg of total polyphenols and 134 mg of total flavonoids) for four weeks on oxidative stress and inflammation after intense exercise in healthy and physically-active male. The authors observed a significant reduction in plasma levels of IL-6, malondialdehyde along with decreased oxidative stress and increased antioxidant capacity, and glutathione levels in the group supplemented with propolis. In the present study, we did not assess biochemical markers of inflammation or oxidative stress, which may be considered a limitation. However, the antioxidant effects of propolis extract have been previously demosntrated by Diniz et al. [34] in healthy volunteers at dosages of 375 and 750 mg per day, supporting the existing literature on its antioxidant properties.

In the present study, we observed that eccentric exercise significantly impaired muscle strength, with notable reductions in muscle force at 2 h, 24 h, 48 h, and 72 h post-exercise, confirming that the eccentric exercise protocol effectively induced muscle damage [54]. However, supplementation with propolis extract optimized muscle strength recovery 2 h after eccentric exercise. These findings align with a previous study [55] that evaluated the effects of one-week supplementation with Brazilian green propolis extract (containing 97.32 mg of artepillin C) and reported improved maximal voluntary isometric contraction of the knee extensors immediately following an exercise fatigue protocol (i.e. 100 maximal voluntary concentric contractions). Recent literature suggests that the probable mechanism responsible for the reduction of ROS and inflammatory activity through propolis extract supplementation likely involve the inhibition of the NF-κB pathway and inflammasome activation [23,49], as well as the enhancement of endogenous antioxidant enzyme activity [17] and the direct antioxidant effects of phenolic compounds [50]. These mechanisms may collectively contribute to mitigating the decline in MVIC, particularly in the initial hours following exercise-induced muscle damage (EIMD).

Additionally, echo intensity was assessed to evaluate the effects of propolis extract supplementation on muscle morphology following EIMD. In the present study, eccentric exercise significantly increased EI, with significant increases observed 72 h post-exercise in placebo group. Our findings align with a previous study indicating that exercise-induced increases in EI are associated with muscle soreness, reduced muscle strength, and diminished muscle quality [53] and marks of muscle inflammation [56]. However, propolis extract supplementation attenuated the rise in EI 2 h post-exercise compared to the placebo group. This finding coincided with enhanced muscle strength recovery at the same time point, further supporting the protective effects of propolis extract on the functionality of contractile units within skeletal muscle tissue. The most pronounced protective effects on muscle morphology were observed between 24 h and 72 h post-EIMD, a timeframe corresponding to the delayed phase of muscle damage characterized by a transient inflammatory response and elevated levels of pro-inflammatory cytokines such as IL-1β, IL-6, IL-8, and TNF-α [6]. These findings suggest that propolis may mitigate oxidative stress and pro-inflammatory cytokine levels following strenuous exercise, as previously demonstrated in human studies [24,25] and animal models [49]. However, a limitation of this study is that the analysis was restricted to selected muscle regions, which may not fully represent systemic or whole-muscle group responses.

In the present study, an increase in muscle thickness was observed 2 h after eccentric exercise only in the control group, suggesting exercise-induced muscle swelling. The increase in muscle thickness after EIMD is likely related to the inflammatory response [57], which involves the recruitment of immune cells (e.g. acute-phase proteins, cytokines, leukocytes, and lymphocytes) and their accumulation at the injury site, coupled with increased muscle membrane permeability [5]. A previous study has shown that propolis can reduce paw edema in mice, decrease tissue infiltrates, and minimize the area of the injured tissue [58]. Additionally, animal models have demonstrated that propolis extract administration reduces edema in induced granuloma [59] and paw edema [60] due to their anti-inflammatory and immunomodulatory activity. These findings align with our results, where the propolis extract supplementation appeared to prevent exercise-induced muscle swelling 2 h after eccentric exercise.

In this context, a significant increase in delayed onset muscle soreness (DOMS) was observed at 24 h and 72 h after eccentric exercise. However, propolis extract supplementation was effective in alleviating muscle soreness in the following days of eccentric exercise. These findings are consistent with a previous study [37], which demonstrated a reduction in DOMS and inflammation, as indicated by decreased lymphocytes levels, after 30 days of supplementation with 70 mg of artepillin C-rich propolis extract in healthy males following intense exercise training. Muscle soreness is closely associated with the inflammatory response induced by muscle damage from eccentric exercise [2]. Our results align with these findings, highlighting the potential of propolis to alleviate muscle soreness, possibly due to its anti-inflammatory properties.

This study has several limitations that should be acknowledged. First, the sample consisted exclusively of trained females, which may limit the generalizability of the results to males or untrained populations. Second, oxidative stress markers were not directly measured, which restricts conclusions regarding the antioxidant effects of EPP-AF supplementation. Third, the analysis of muscle damage was limited to selected muscle regions assessed via ultrasound, which may not fully represent systemic or whole-muscle group responses. Future research should address these limitations by including more diverse participant groups, incorporating direct biochemical markers of oxidative stress, and expanding muscle assessment methods to provide a more comprehensive understanding of propolis extract’s effects on exercise-induced muscle damage.

## 5. Conclusion

In this study, the eccentric exercise protocol resulted in significant impairments in muscle strength and morphological muscle quality, accompanied by increased muscle soreness and swelling. Propolis extract supplementation improved muscle strength recovery, preserved morphological muscle quality, and alleviated muscle soreness in the days following exercise-induced muscle damage. The protective effects varied across measures, with the most significant improvements observed in muscle soreness and morphological quality during the later inflammatory phase. These findings suggest that propolis supplementation may contribute to accelerated recovery and mitigation of initial muscle damage. Overall, the bioactive compounds present in Brazilian green propolis extract show potential for alleviating symptoms of muscle damage, offering benefits for physically active females and individuals engaging in intense or unaccustomed exercise.

## Abbreviations

ANOVA: Analysis of variance
DOMS: Delayed onset muscle soreness
EI: Echo intensity
EIMD: Exercise-induced muscle damage
EPP-AF: Standardized Brazilian green propolis extract
MQ_m_: Morphological muscle quality
MVIT: Maximal voluntary isokinetic torque
MT: Muscle thickness
PLA: Placebo
ROS: Reactive oxygen species
VAS: Visual analogic scale
VO₂ max: Maximal oxygen consumption
