# Correction

**DOI:** 10.1080/15502783.2025.2577011

**Published:** 2025-10-27

**Article title**: Artepillin C-rich propolis extract supplementation promotes muscle

recovery following exercise-induced muscle damage in resistance-trained

young females: a randomized, placebo-controlled trial

**Authors**: Ramos Junior, O. J. F., Veiga, N. S., Berretta, A. A., & Alvares, T. S.

**Journal**: *Journal of the International Society of Sports Nutrition*

**DOI**: https://doi.org/10.1080/15502783.2025.2569908

This article was published earlier without ‘Figure 3’. This has now been included and republished in the original article. The below mentioned is the Figure 3 which is now included in the original article.

**Figure 3.**
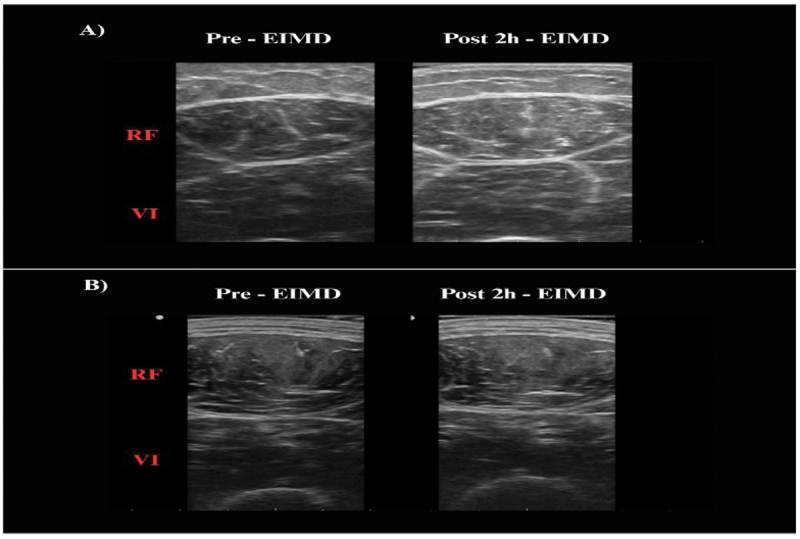
Representative ultrasound images and echo intensity measurements. Images were taken before and 2 hours after EIMD for the PLA group (A) and the EPP-AF group (B). RF = rectus femoris muscle; VI = vastus intermedius muscle.

